# One Year of Endurance Exercise Training Does not Improve Vascular Function in Non‐Trained Limbs in Healthy Individuals

**DOI:** 10.1111/sms.70354

**Published:** 2026-08-21

**Authors:** Marcos Paulo Rocha, Casper Sejersen, Mads Fischer, Andrea Tamariz‐Ellemann, Thomas Bonne, Jacob Bejder, Carsten Lundby, Nikolai B. Nordsborg, Lars Nybo, Jakob Solgaard Jensen, Lasse Gliemann

**Affiliations:** ^1^ The August Krogh Section for Human Physiology, Department of Nutrition, Exercise and Sports University of Copenhagen Copenhagen Denmark

**Keywords:** cardiovascular health, endurance exercise training, vascular function

## Abstract

Regular endurance exercise is widely recognized for its cardiovascular health benefits, with improvements in vascular function particularly relevant for populations with impaired vascular health. However, it is unknown whether prolonged adherence to endurance exercise training in sedentary but healthy young individuals can improve vascular function in non‐active limbs. This study investigated the impact of 1 year of supervised endurance exercise on vascular function in previously untrained, healthy young participants. Twelve healthy young adults (25 ± 4 years) were included in a 12‐month supervised cycling program, during which maximal aerobic capacity (V̇O_2_max) and vascular function were assessed at baseline and after 1, 4, 6, and 12 months of training. Forearm vascular function was assessed using intra‐arterial infusions of acetylcholine (ACh; endothelium‐dependent vasodilation) and sodium nitroprusside (SNP; endothelium‐independent vasodilation) and flow‐mediated dilation (FMD). Seven participants completed the 12‐month protocol and showed no change in endothelium‐dependent or independent vasodilation assessment at any time point during the 12‐month training period (*p* > 0.05), despite a substantial increase in aerobic capacity during the first 6 months of training (baseline: 40.3 vs. 6 months: 50.8 mL·min^−1^·kg^−1^, *p* = 0.001), with no further change observed from 6 to 12 months (51.3 mL·min^−1^·kg^−1^, *p* > 0.05). One year of endurance exercise training improved aerobic capacity without improving vascular function in non‐trained limbs in previously untrained but healthy individuals. These findings support the idea that vascular adaptations to endurance training may reflect a region‐specific training effect with vascular remodeling confined to actively engaged limbs and, thus, undetectable in vascular territories not directly involved in exercise training.

## 1 Introduction

Regular exercise has long been recognized as an effective nonpharmacological strategy for promoting cardiovascular health due to its numerous cardioprotective benefits [1]. Among the various cardiovascular adaptations induced by regular exercise, improvements in vascular function are particularly important for individuals at elevated cardiovascular risk [2, 3, 4]. Vascular endothelial dysfunction serves as an early marker of cardiovascular disease, preceding the development of conditions such as coronary artery disease, hypertension, and stroke, and contributes to both disease progression and adverse cardiovascular events [5].

Endothelial cells play a critical role in maintaining vascular homeostasis and modulating the activity of vascular smooth muscle cells (VSMCs), inducing either vasoconstriction or vasodilation depending on the stimulus. Together with the sympathetic nervous system, the endothelium regulates vascular tone through mediators such as nitric oxide (NO), prostacyclins, prostaglandins, thromboxane, angiotensin II, and endothelin‐1. In addition, adenosine triphosphate (ATP) and adenosine can activate endothelial receptors and stimulate the release of NO and prostacyclin [6]. Exercise induces a systemic hemodynamic response, and although the largest increases in blood flow and endothelial shear stress occur in the vasculature of engaged limbs during exercise [7, 8], these hemodynamic stimuli are not restricted to the exercising limbs and may also be observed in non‐engaged limbs [9]. Consequently, the vascular effects of exercise are not restricted to the vascular beds of the engaged muscles but instead encompass the entire vascular system [3, 10, 11]. With repeated exposure to exercise‐induced shear stress, the vasculature undergoes progressive adaptive changes, resulting in chronic improvements in vascular function, through mechanisms including upregulation of endothelial nitric oxide synthase (eNOS) expression and enhanced nitric oxide (NO) bioavailability, as observed in populations with cardiovascular disease [12]. These vascular improvements are attributed mainly to the sustained increases in shear stress that act as a mechanotransductive stimulus for endothelial adaptation [13].

Endurance exercise training significantly reduces cardiovascular risk, eliciting beneficial systemic vascular adaptations beyond the active limbs [3, 14, 15], positioning endurance exercise as a cornerstone in preventing and managing cardiovascular diseases [16]. However, it remains unclear whether endurance training can further optimize vascular health in non‐exercising limbs of otherwise healthy individuals, thereby expanding the functional margin between normal healthy vascular function and impaired vascular function. Previous exercise training intervention studies have produced conflicting results regarding improvements in vascular function between trained and non‐trained limbs. Although studies assessing vascular function in exercise‐trained limbs consistently report improvements [17, 18, 19], findings are less consistent when vascular function is measured in non‐trained limbs. Some studies report significant improvements in non‐trained limbs following exercise training in untrained but otherwise healthy individuals [3, 20], whereas other studies have found no such effect [21, 22]. Thus, the extent to which exercise training elicits systemic vascular adaptations beyond the engaged skeletal muscles remains controversial. These discrepancies may stem from whether the vascular beds assessed were somewhat stimulated in the exercise modality or from methodological differences in the assessment of vascular function, where some studies assess flow‐mediated dilation (FMD), whereas others use intra‐arterial infusion of vasoactive compounds. However, a common feature of these studies is their relatively short intervention durations, ranging from 4 to 10 weeks. It is likely that a significantly longer duration of the training intervention would lead to solid adaptations in vascular function beyond the exercised limbs. Therefore, this study aimed to assess vascular function in non‐trained limbs, in previously untrained, but healthy young individuals following 1 year of supervised endurance training, with an evaluation of vascular function at multiple time points throughout the year using both FMD and intra‐arterial infusions. This approach enabled the assessment of both short‐ and long‐term effects of endurance exercise training on the vascular function of non‐engaged limbs. We hypothesized that prolonged endurance training would lead to improvements in vascular function of non‐trained limbs in healthy, untrained young individuals.

## 2 Methods

### 2.1 Ethical Approval

The study was approved by the Danish National Committee on Health Research Ethics, Capital Region of Copenhagen (H‐21011041), and conducted following the latest guidelines of the Declaration of Helsinki. Before participation, all subjects received detailed oral and written explanations of the experimental procedures and provided signed informed consent.

### 2.2 Subjects

Participants were recruited through advertisements on various media platforms and invited to perform supervised exercise cycling training for 12 months. Twelve healthy young subjects (seven males and five females; 25 ± 5.2 years, 74.8 ± 14.6 kg), non‐smokers without chronic diseases who had never participated in competitive endurance sports and had not engaged in regular exercise training (at least 3 times per week) for a minimum of 6 months before the start of the project, with a V̇O_2_max < 45 mL·min^−1^·kg^−1^ for males and < 40 mL·min^−1^·kg^−1^ for females, were included. Subjects' characteristics are presented in Table 1.

**TABLE 1:** Subjects' characteristics.

*N* (F/M)	7 (3/4)
Age (years)	25 ± 4
Height (m)	1.78 ± 0.1
Weight (kg)	74.6 ± 14.5
BMI (kg/m^2^)	23.4 ± 3.1
BSA (m^2^)	1.9 ± 0.2
FFM in body (kg)	56 ± 13.9
V̇O_2_max (L/min)	3.05 ± 1.0

Abbreviations: BSA, body surface area; FFM, fat‐free mass; kg, kilograms; kg/m^2^, kilograms per square meter; L/min, liter per minute; m, meters; *N*, number of subjects; V̇O_2_max, maximum oxygen uptake.

Of the 12 enrolled participants, five volunteers did not complete the training (three dropouts, one pregnancy, and one unsuccessful catheter placement). Consequently, seven subjects (four males and three females; age: 25 ± 4 years, 74.6 ± 14.5 kg, BMI 23.4 ± 3.1) completed the full 1‐year supervised cycling training and were included in the analysis.

### 2.3 Study Design

A longitudinal study design was employed where participants engaged in supervised endurance exercise training for 12 months. Maximal aerobic capacity (V̇O_2_max) and vascular function were assessed at five points: the initial visit (baseline) and after 1, 4, 6, and 12 months of endurance exercise training. Vascular function was evaluated during separate visits from the V̇O_2_max testing day, with a minimum interval of 4 days between these visits. Participants were instructed to abstain from exercise, alcohol, and caffeine‐containing beverages for 24 h prior to each laboratory visit.

### 2.4 Maximal Aerobic Capacity (V̇O_2_max) Test

The V̇O_2_max was determined using an incremental exercise test on a cycle ergometer (Lode Excalibur, Groningen, Netherlands), and pulmonary gas exchange was assessed with a mixing chamber gas analyzer system (Quark, CPET, Cosmed, Italy). The gas analyzer system was calibrated according to the manufacturer's guidelines before each test. Prior to the V̇O_2_max test, subjects had performed a Maximal Fat Oxidation (MFO) test that served as a warm‐up. A description of MFO is presented in Supporting Information, but data are not included in this paper. Male subjects began cycling at 90 W, whereas females started at 75 W. The workload increased by 25 W every minute until exhaustion. The V̇O_2_max was defined as the highest value of oxygen consumption averaged over a 30‐s interval, recorded during the test and validated as previously reported. After the V̇O_2_max test, the equation: Watt_max_ = *W*_compl_ + 25 · (*t*·60^−1^) was used to calculate the maximal power output (Watt_max_). The “*W*_compl_” represents the last completed workload, and “*t*” represents the total duration time at the final workload stage. Participants also performed a 30‐min time trial (TT) test to prescribe exercise intensity for the training session. Details of the 30‐min TT test can be found in Supporting Information.

### 2.5 Supervised Exercise Cycling Training Protocol

The training sessions were conducted at a fitness center at the University of Copenhagen, where participants were instructed to attend three times a week for 60 min to perform supervised cycling sessions on stationary bikes (Body Bike Supreme, Body Bike). Training intensity was prescribed using individualized power outputs (watts) from the Watt_max_ test, initially based on 30‐min TT, and later derived from monthly performance tests using a 2‐parameter Critical Power model. Training zones (Z1–Z6) were calculated in TrainingPeaks (TrainingPeaks LLC, Boulder, CO, USA) relative to the individual critical power. Training zones are described in detail in Supporting Information. Sessions progressed from 30 to 45 min at 75%–95% critical power in the initial phase to 1–2 h, including intervals at 95%–130% CP, with a periodized structure targeting performance peaks during test weeks. Training was monitored via pedal‐based power meters (Assioma UNO; Favero Electronics, Arcade, Italy) and heart rate sensors, with data analyzed using TrainingPeaks. Training load was quantified as total energy expenditure (kJ), calculated from average power output and duration. For more details, see Supporting Information.

### 2.6 Vascular Function Assessment Protocols

Vascular function assessments were carried out using both invasive and non‐invasive methods. Before each visit, body composition was assessed using whole‐body dual‐energy X‐ray absorptiometry scanning (Lunar iDXA, Encore software 18; GE Healthcare, USA). Total right arm mass was used to normalize the infusion rates for acetylcholine (ACh) and sodium nitroprusside (SNP). Female participants attended the lab during the same phase of their menstrual cycle to standardize testing conditions and reduce potential variability related to hormonal fluctuations [23, 24]. Hormonal levels were not directly measured, and it was not possible to schedule testing in the early follicular phase in all participants, but the timing of the first visit was recorded and repeated at the following visits. Subjects were positioned supine, and local anesthesia (lidocaine, 20 mg·mL^−1^; AstraZeneca, Denmark) was administered subcutaneously superficial to the right brachial artery at the fossa cubiti, followed by the insertion of a catheter (20‐gauge flow‐switch, Becton Dickinson Infusion Therapy System, UT, USA) in the right brachial artery and an antecubital vein (Venflon, 20‐gauge; Becton Dickinson Infusion Therapy System, UT, USA) for simultaneous and continuous intra‐arterial and intra‐venous pressure recordings (Truwave; Edwards Lifescience, Irvine, CA). A three‐way stopcock connector (Connecta; Becton Dickinson Infusion Therapy Systems, UT, USA) was used for concurrent blood pressure measurement and drug infusion. Intra‐vascular blood pressure was obtained by a pressure transducer connected to the catheters (pressure monitoring set; Edwards Lifescience, Irvine, CA). Data were recorded and stored in Lab Chart 8 (AD Instruments, Sydney, Australia). A 15‐min recovery period was allowed between assessments.

### 2.7 Endothelium‐Dependent and Smooth Muscle Vascular Assessment

**FIGURE 1:**
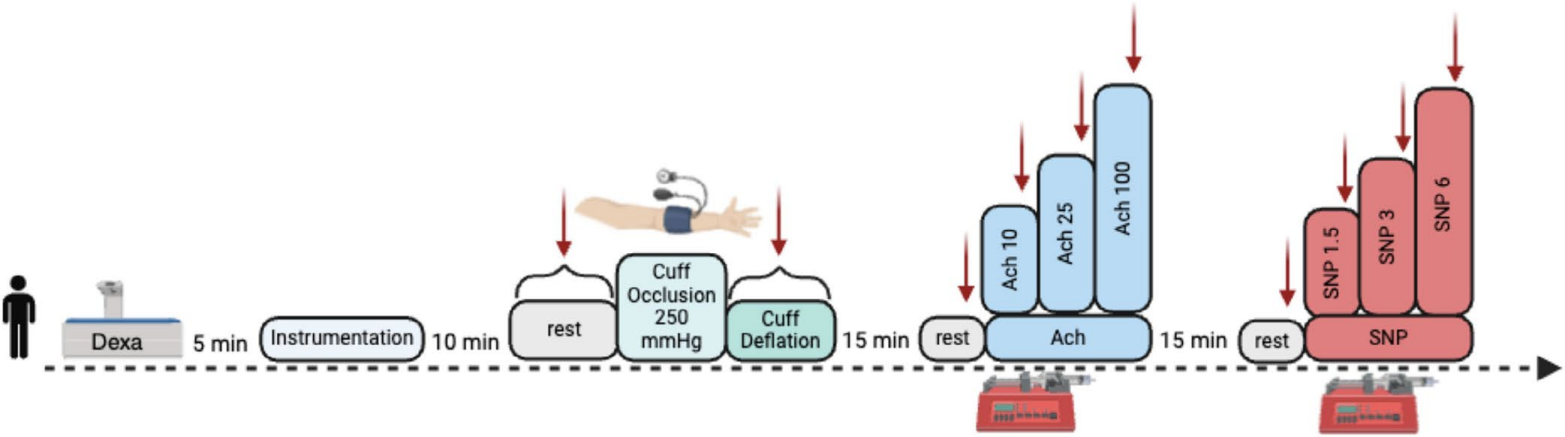
Illustrative figure of the experimental timeline procedures. Dexa, whole‐body dual‐energy X‐ray absorptiometry scanning. ACh, acetylcholine; FMD, flow‐mediated dilation; SNP, sodium nitroprusside. The red arrows represent the moments for blood flow measurement taken during the last 30 s of each infusion rate. ACh and SNP infusion rates were normalized based on total arm mass.

Endothelium‐dependent vascular function was assessed using an intra‐arterial infusion of ACh with a cumulative increase in the infusion rates of 10 (ACh1), 25 (ACh2), and 100 (ACh3) μg·min^−1^·kg^−1^ (normalized to total arm mass^−1^; miochol‐E; Bausch & Lomb Inc., Bridgewater, NJ) [25]. For smooth muscle assessment, an intra‐arterial infusion of the NO donor, SNP, was used with a cumulative increase in the infusion rates of 1.5 (SNP1), 3 (SNP2), and 6 (SNP3) μg·min^−1^·kg^−1^ (normalized to total arm mass^−1^; Hospira Inc., Lake Forest, IL). Each dose was infused for 2.5 min, and at the last 30 s, all blood flow and blood pressure were simultaneously measured for analysis of vascular conductance (Figure 1).

### 2.8 Flow‐Mediated Dilation (FMD) Protocol

Following guidelines for FMD measurement [26], a rapid inflation/deflation pneumatic cuff system (E‐20 Rapid Cuff Inflator; D.E. Hokanson, USA) was positioned around the forearm, just below the olecranon process. The left arm (the opposite of the catheter arm) was used for all participants. After 20 min of quiet resting, the arterial diameter and blood velocity baseline were recorded for 1 min as a baseline measurement, followed by rapid cuff inflation to a pressure of 250 mmHg, which was maintained for 5 min until rapid cuff deflation occurred. Recordings for reactive hyperaemia began 10 s before the cuff was deflated and continued for 3 min [26]. Data were exported and analyzed offline using wall‐tracking software (Vascular Research Tools 7; Medical Imaging Applications LLC; Coralville, IA), which operates independently of the user. The region of interest for arterial diameter analysis was selected based on image quality, which revealed a distinct boundary between the artery walls and the lumen, characterized by clear pignoli lines. The FMD was calculated using Equation (3). The resting diameter was calculated as the average of over 60 s. After the cuff deflation, the peak diameter was the highest diameter achieved within the 3‐min video recording. The shear rate stimulus was calculated using Equation (4) in the period from the moment the cuff pressure was released until the moment of reaching the peak diameter [27]. The time to peak was calculated from when the cuff was deflated until the diameter reached its peak value.

### 2.9 Brachial Arterial Images

A linear probe (L‐9) operating at a Doppler frequency of 5.6 MHz and an imaging frequency of 12.0 MHz was positioned 2–4 cm distal of the olecranon (GE Vivid E9; GE Healthcare, Chicago, Illinois, USA). The angle correction of 60° or below was respected for all images. The B‐mode (2D images) ultrasound was used to locate the optimal artery image in the arm with visible pignoli lines.

The brachial artery was then recorded in B‐mode with ECG coupling for R‐wave tracking for subsequent analysis. Doppler files were exported through the ECHOpac system (software version 203; GE Healthcare). The arterial diameter analysis was performed using automated edge detection and wall‐tracking software (Vascular Research Tools 7; Medical Imaging Applications, Coralville, IA, USA).

(1) $$\text{Blood flow}:\mathrm{BV}\left(\mathrm{cm}\cdot s^{-1}\right)\cdot \pi \cdot {\left(\frac{\text{diameter}\left(\mathrm{cm}\right)}{2}\right)}^{2}\cdot 60\left(s\right).$$

(2) $$\text{Vascular conductance}:\mathrm{VCT}=\frac{\mathrm{BF}\;\left(\mathrm{mL}\cdot \min^{-1}\right)}{\mathrm{MAP}\;\left(\text{mmHg}\right)-\text{venous pressure}\;(\mathrm{mmHg})}$$

(3) $$\mathrm{FMD}\;\left(\%\right):\frac{\left(\text{Peak diameter}\;\left(\mathrm{cm}\right)-\text{Baseline diameter}\;\left(\mathrm{cm}\right)\right)}{\text{Baseline diameter}\;\left(\mathrm{cm}\right)}\times 100$$

(4) $$\text{Shear rate}:\text{Shear rate}\;\left(s^{-1}\right)=4\cdot \left(\frac{\mathrm{BV}\left(\mathrm{cm}\cdot s^{-1}\right)}{\text{diameter}\;\left(\mathrm{cm}\right)}\right)$$

BV, blood velocity; VCT, vascular conductance; MAP, mean arterial pressure.

### 2.10 Statistical Analysis

Data normality was assessed using the Shapiro–Wilk test. A mixed‐effects model was employed to determine the impact of endurance exercise training on vascular function with time points (visits) and infusion rates as fixed effects and subjects as random effects. The estimated marginal mean (EMM) post hoc analysis using Tukey adjustment for multiple comparisons was employed to identify significant differences in data points. The graphic illustration was created using GraphPad Prism (Version 10.4.1). Results are expressed as mean ± SD, with statistical significance accepted at *p* < 0.05. Statistical analysis was performed using RStudio (version 2024.12.0).

## 3 Results

### 3.1 Maximal Aerobic Capacity (V̇O_2_max)

Supervised endurance exercise training increased subjects' aerobic capacity, with a 26.7% rise in V̇O_2_max after 6 months (baseline: 40.3 vs. 6 months: 50.8 mL·min^−1^·kg^−1^, *p* = 0.001). No difference was observed between 6 and 12 months of endurance training (51.3 mL·min^−1^·kg^−1^, *p* > 0.05).

### 3.2 Flow‐Mediated Dilation (FMD)

**FIGURE 2:**
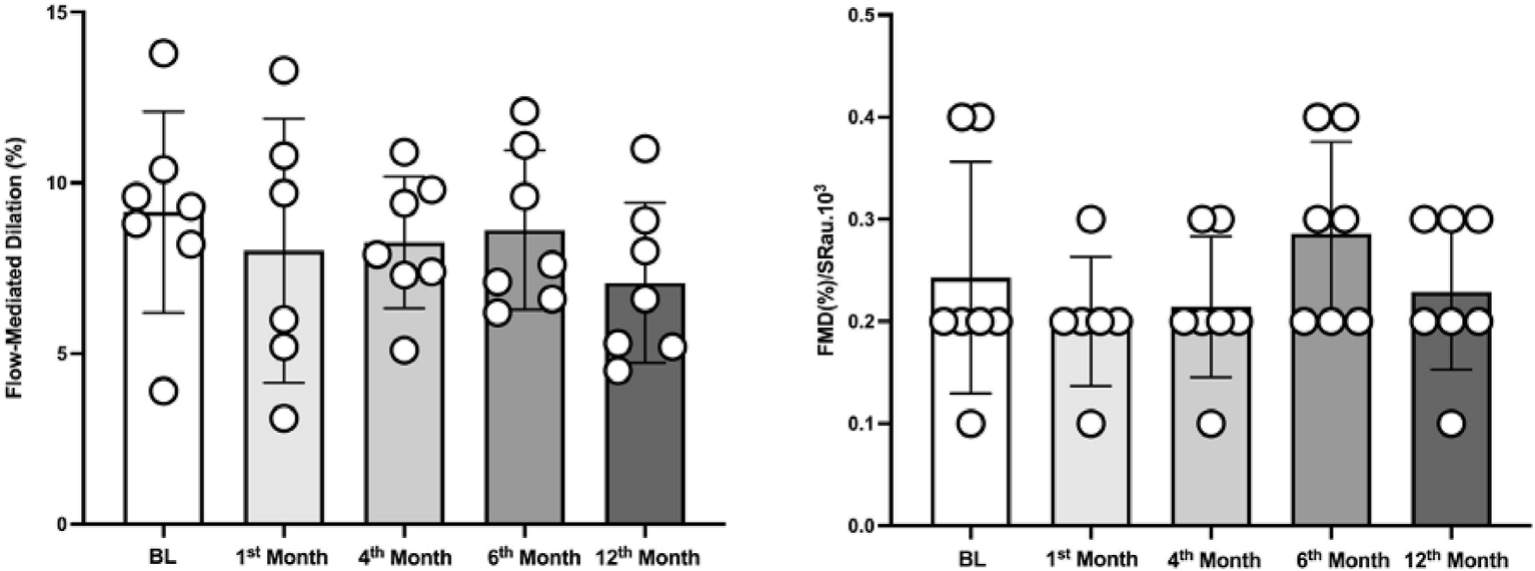
Flow‐mediated dilation (FMD) percentage response for all five visits, and FMD/SRauc, flow‐mediated dilation divided by shear rate area under the curve. BL, baseline.

Arterial blood pressure remained stable during the FMD protocol (Table 2). The diameter of the brachial artery measured at rest was not different between time points (*p* > 0.05). The FMD response, assessed after 5 min of ischemia, revealed no significant improvement after 12 months of exercise training (*p* > 0.05) (Figure 2).

**TABLE 2:** FMD parameters at five different time points during 1 year of endurance exercise training.

	Baseline	1st month	4th month	6th month	12th month
Brachial artery					
Rest diameter (mm)	3.67 ± 0.63	3.43 ± 0.56	3.67 ± 0.65	3.66 ± 0.60	3.60 ± 0.57
Peak diameter (mm)	4.00 ± 0.65	3.68 ± 0.53	3.98 ± 0.72	3.97 ± 0.66	3.86 ± 0.65
Absolute change (mm)	0.33 ± 0.09	0.26 ± 0.12	0.30 ± 0.09	0.31 ± 0.10	0.26 ± 0.11
SR_auc_ (AUC)	39.2 ± 6.91	44.2 ± 10.9	39.3 ± 8.35	31.9 ± 7.58	33.4 ± 5.20

*Note:* Data are presented as mean ± SD.

Abbreviations: FMD, flow‐mediated dilation; SR AUC to peak, shear rate area under curve to peak.

### 3.3 Acetylcholine and Sodium Nitroprusside Infusions

#### 3.3.1 Acetylcholine Infusion

Brachial blood flow increased significantly in response to ACh infusion in a dose‐dependent manner across all infusion rates compared during all visits, except for the ACh1 infusion rate at the 1‐month visit and 12‐month visit (*p* = 0.79 and *p* = 0.47, respectively). Overall, the analysis revealed a significant main effect of time (*p* = 0.01) and infusion rate (*p* < 0.001). However, there was no significant difference in the ACh‐induced blood flow response between baseline and subsequent visits.

Intra‐arterial administration of ACh did not change systolic BP at any of the three infusion rates (*p* > 0.05). However, at the 1‐month visit, diastolic BP showed an increase at the high infusion rate of ACh3 (100 μg·min^−1^·kg^−1^) compared to rest (∆ 23 ± 26, *p* < 0.001) and ACh1 infusion rate (10 μg·L·min^−1^) (∆ 22 ± 26, *p* < 0.001). The increase in diastolic blood pressure response induced a rise in mean arterial pressure at ACh3 infusion rate compared to rest (∆ 20 ± 24, *p* = 0.003) and ACh1 infusion rate (∆ 20 ± 28, *p* = 0.002).

The higher ACh infusion rate (ACh3) resulted in increased vascular conductance (mL·min^−1^·mmHg^−1^) at all time points throughout the intervention period (*p* < 0.05). Yet, at the first and 12‐month visits, the ACh2 infusion rate increased VC above rest values (Table 3).

**TABLE 3:** Cardiovascular and hemodynamic responses at rest and during the infusion of three doses of ACh in the infused forearm at five different time points during 1 year of endurance exercise training.

	Baseline	1st month	4th month	6th month	12th month
SBP (mmHg)					
Rest	129 ± 8.9	123 ± 11.8	128 ± 6.5	123 ± 7.8	125 ± 12.0
ACh1	116 ± 7.3	116 ± 10.6	117 ± 9.6	115 ± 8.7	119 ± 10.0
ACh2	115 ± 8.3	118 ± 12.7	116 ± 11.0	114 ± 8.5	119 ± 11.0
ACh3	118 ± 9.5	130 ± 28.3	116 ± 12.0	114 ± 8.3	124 ± 15.4
DBP (mmHg)					
Rest	69 ± 7.3	69 ± 6.5	68 ± 4.5	64 ± 3.9	68 ± 8.3
ACh1	69 ± 5.8	70 ± 7.2	67 ± 5.5	65 ± 3.6	68 ± 8.1
ACh2	69 ± 7.3	73 ± 9.0	67 ± 5.5	65 ± 3.6	70 ± 9.3
ACh3	73 ± 2.9	92 ± 33.0^†‡^	74 ± 8.0	73 ± 6.1	78 ± 11.2
MAP (mmHg)					
Rest	89.0 ± 7.4	87.5 ± 7.7	87.4 ± 5.3	83.7 ± 4.9	87.1 ± 9.4
ACh1	86.4 ± 6.1	86.9 ± 7.8	85.3 ± 6.5	82.0 ± 4.6	85.9 ± 9.6
ACh2	86.2 ± 7.8	90.2 ± 9.7	85.1 ± 6.9	82.2 ± 4.7	87.3 ± 10.4
ACh3	90.1 ± 4.0	107.1 ± 31.5	89.9 ± 9.4	86.6 ± 6.2	94.7 ± 12.8
Diameter (cm)					
Rest	0.37 ± 0.08	0.35 ± 0.07	0.35 ± 0.06	0.36 ± 0.06	0.36 ± 0.09
ACh1	0.40 ± 0.07	0.37 ± 0.07	0.39 ± 0.07	0.38 ± 0.06	0.38 ± 0.08
ACh2	0.41 ± 0.06	0.39 ± 0.07	0.39 ± 0.07^†^	0.38 ± 0.06	0.37 ± 0.07
ACh3	0.42 ± 0.07^†^	0.39 ± 0.07	0.40 ± 0.06^†^	0.41 ± 0.06^†^	0.38 ± 0.07
Velocity (cm·s^−1^)					
Rest	10.30 ± 5.7	10.16 ± 6.3	10.30 ± 3.4	10.87 ± 9.0	8.42 ± 6.2
ACh1	42.75 ± 6.4^†^	38.07 ± 7.3^†^	38.50 ± 3.5^†^	35.28 ± 7.2^†^	30.74 ± 16.6^†^
ACh2	47.48 ± 7.8^†^	46.40 ± 7.8^†^	44.03 ± 7.5^†^	40.85 ± 10.1^†^	39.94 ± 17.2^†^
ACh3	64.01 ± 14.8^†‡^	70.83 ± 15.3^†‡§^	64.83 ± 14.8^†‡§^	60.22 ± 10.7^†‡§^	55.59 ± 22.0^†‡^
Blood flow (mL·min^−1^)					
Rest	75.33 ± 62	54.91 ± 22	60.62 ± 27	77.72 ± 97	65.56 ± 87
ACh1	339.51 ± 140^†^	258.77 ± 105	285.31 ± 124^†^	259.15 ± 129^†^	226.29 ± 154
ACh2	378.24 ± 150^†^	335.96 ± 140^†^	326.75 ± 142^†^	298.14 ± 147^†^	271.91 ± 151^†^
ACh3	525.83 ± 198^†‡^	550.98 ± 277^†‡^	504.03 ± 170^†‡^	488.13 ± 212^†‡^	425.86 ± 243^†‡^
VC (mL·min^−1^·mmHg^−1^)					
Rest	1.0 ± 0.9	0.7 ± 0.3	0.8 ± 0.4	1.3 ± 1.6	1.2 ± 1.5
ACh1	4.6 ± 2.0	3.5 ± 1.5	3.9 ± 1.9	4.0 ± 1.9	4.0 ± 3.1
ACh2	5.1 ± 2.1^†^	4.3 ± 1.8	4.5 ± 2.3	4.7 ± 2.3	5.4 ± 5.1^†^
ACh3	6.9 ± 2.9^†^	6.8 ± 4.8^†^	6.5 ± 2.1^†^	7.1 ± 3.0^†^	7.0 ± 5.2^†^

*Note:* ACh1, 10 μg·min^−1^·kg^−1^; ACh2, 25 μg·min^1^·kg^1^; ACh3, 100 μg·min^−1^·kg^−1^. Data are presented as mean ± SD. ^†^ *p* < 0.05 for difference between rest, ^‡^ *p* < 0.05 for difference between 10 μg·min^−1^·kg^−1^, and ^§^ *p* < 0.05 for difference between 25 μg·min^−1^·kg^−1^.

Abbreviations: DBP, diastolic blood pressure; MAP, mean arterial pressure; SBP, systolic blood pressure; VC, vascular conductance.

#### 3.3.2 Sodium Nitroprusside Infusion

Brachial blood flow increased significantly in response to SNP infusion compared to rest at all visits (Table 4). Specifically, the SNP2 and SNP3 infusion rates consistently elevated blood flow at all time points, whereas SNP1 increased blood flow at the 1‐ and 4‐month visits (*p* = 0.01 and *p* = 0.002, respectively). However, no significant differences in blood flow were observed between visits for any infusion rate. A significant main effect of time (*p* = 0.01) and infusion rate (*p* < 0.001) was found, but there was no interaction between visits and infusion rates (*p* = 0.87).

**TABLE 4:** Cardiovascular and hemodynamic responses at rest and during the infusion of three doses of SNP in the infused forearm at five different time points during 1 year of endurance exercise training.

	Baseline	1st month	4th month	6th month	12th month
SBP (mmHg)					
Rest	130 ± 9.8	127 ± 14.0	128 ± 9.8	126 ± 10.7	130 ± 12.0
SNP1	119 ± 7.3^†^	113 ± 16.8^†^	116 ± 14.0^†^	114 ± 8.6^†^	120 ± 13.2
SNP2	115 ± 8.5^†^	113 ± 12.1^†^	113 ± 12.8^†^	112 ± 7.9^†^	116 ± 12.6^†^
SNP3	114 ± 7.9^†^	112 ± 12.8^†^	111 ± 12.4^†^	111 ± 7.4^†^	117 ± 13.0^†^
DBP (mmHg)					
Rest	69 ± 3.7	70 ± 7.1	69 ± 6.6	66 ± 4.6	70 ± 8.6
SNP1	68 ± 3.6	68 ± 10.1	67 ± 8.7	65 ± 5.0	70 ± 9.0
SNP2	67 ± 4.9	69 ± 7.9	66 ± 6.7	64 ± 4.0	67 ± 7.7
SNP3	67 ± 5.5	69 ± 9.1	64 ± 5.9	65 ± 5.3	68 ± 6.5
MAP (mmHg)					
Rest	90.0 ± 4.4	89.3 ± 9.1	89.2 ± 8.6	86.3 ± 6.2	90.4 ± 10.0
SNP1	86.6 ± 4.3	84.9 ± 11.7	85.0 ± 10.4	81.6 ± 5.9	87.2 ± 11.0
SNP2	84.5 ± 5.8	85.3 ± 8.8	85.8 ± 8.5	80.6 ± 4.9	84.2 ± 9.8
SNP3	84.2 ± 5.2	85.2 ± 9.4	80.9 ± 8.2	80.7 ± 5.2	84.6 ± 8.4
Diameter (cm)					
Rest	0.37 ± 0.08	0.36 ± 0.06	0.37 ± 0.06	0.36 ± 0.06	0.37 ± 0.08
SNP1	0.38 ± 0.08	0.39 ± 0.06	0.38 ± 0.07	0.37 ± 0.06	0.39 ± 0.09
SNP2	0.39 ± 0.08	0.39 ± 0.06	0.40 ± 0.07	0.38 ± 0.05	0.39 ± 0.09
SNP3	0.40 ± 0.07	0.40 ± 0.06	0.41 ± 0.07^†^	0.39 ± 0.06	0.41 ± 0.09
Velocity (cm·s^−1^)					
Rest	14.97 ± 9.4	12.85 ± 4.7	11.38 ± 4.4	12.05 ± 6.2	10.74 ± 4.0
SNP1	29.09 ± 7.4^†^	33.20 ± 2.0^†^	31.11 ± 7.8^†^	26.35 ± 8.5^†^	25.42 ± 6.7^†^
SNP2	32.66 ± 7.8^†^	36.95 ± 6.3^†^	33.28 ± 6.5^†^	28.32 ± 10.2^†^	28.74 ± 8.4^†^
SNP3	37.03 ± 9.2^†^	39.44 ± 7.8^†^	37.67 ± 8.0^†^	32.26 ± 8.6^†^	32.05 ± 9.5^†^
Blood flow (mL·min^−1^)					
Rest	100.05 ± 87	78.88 ± 35	69.61 ± 25	75.20 ± 56	74.65 ± 53
SNP1	217.18 ± 122	239.95 ± 83^†^	221.93 ± 90^†^	178.83 ± 93	183.74 ± 94
SNP2	253.54 ± 150^†^	271.05 ± 106^†^	254.32 ± 94^†^	207.80 ± 129^†^	224.98 ± 140^†^
SNP3	294.71 ± 161^†^	311.88 ± 132^†^	317 ± 137^†^	242.71 ± 117^†^	262.21 ± 148^†^
VC (mL·min^−1^·mmHg^−1^)					
Rest	1.3 ± 1.1	1.0 ± 0.6	0.9 ± 0.3	1.1 ± 0.9	1.3 ± 1.0
SNP1	2.9 ± 1.7	3.3 ± 1.3	3.0 ± 1.4	2.8 ± 1.7	3.6 ± 3.4
SNP2	3.5 ± 2.3	3.6 ± 1.2	3.5 ± 1.4	3.3 ± 2.1	4.4 ± 3.7^†^
SNP3	3.9 ± 1.9	4.2 ± 1.5	4.5 ± 2.0^†^	3.8 ± 1.6	5.0 ± 3.9^†^

*Note:* Data are presented as mean ± SD. ^†^ *p* < 0.05 for difference between rest.

Abbreviations: DBP, diastolic blood pressure; MAP, mean arterial pressure; SBP, systolic blood pressure; SNP1, sodium nitroprusside 1.5 μg·min^−1^·kg^−1^; SNP2, sodium nitroprusside 3 μg·min^−1^·kg^−1^; SNP3, sodium nitroprusside 6 μg·min^−1^·kg^−1^; VC, vascular conductance.

Systolic blood pressure decreased during SNP infusion compared with rest, with significant reductions evident across SNP doses at most time points during the 12‐month endurance exercise training intervention (*p* < 0.05; Table 4). In contrast, diastolic blood pressure and mean arterial pressure remained unchanged at any infusion rate or time point (*p* > 0.05; Table 4). For vascular conductance (mL·min^−1^·mmHg^−1^), the SNP3 infusion rate in the 4th month significantly increased vascular conductance compared to resting (*p* < 0.001). During the 12‐month visit, SNP2 and SNP3 also significantly increased vascular conductance compared to resting (*p* = 0.02 and *p* = 0.001, respectively) (Table 4).

## 4 Discussion

This is the first study to provide insights into the effects of 1 year of supervised endurance exercise training on vascular function in previously untrained but healthy individuals. The main finding was that there were no significant improvements in endothelial‐dependent or independent vascular function in the upper limbs over the 12‐month training period, despite solid improvements in aerobic capacity.

### 4.1 Exercise Training Does not Improve Vascular Function in Non‐Trained Limbs

Contrary to the hypothesis, 1 year of exercise training did not improve vascular function assessed by arterial infusion of ACh in non‐trained limbs in this cohort of healthy participants. The absence of robust changes in endothelium‐dependent vasodilation following 1 year of supervised endurance training suggests that vascular function in limbs not directly involved in the exercise may not substantially adapt in untrained but otherwise healthy individuals, even following a prolonged training period. There are several potential explanations for this lack of training effect. It is possible that the shear stress stimulus generated during cycle exercise training is insufficient to elicit additional vascular adaptations in non‐directly trained limbs of already healthy individuals. In addition, the relatively preserved vascular function of young healthy adults may limit the magnitude of detectable improvement compared with populations exhibiting vascular dysfunction. Thus, baseline vascular status may be an important determinant of the responsiveness to endurance training.

The assessment of VSMC function via SNP infusion showed no improvement throughout the study, indicating that VSMC function in non‐active limbs is not affected by 1 year of aerobic training. Our findings align with previous observations in healthy populations [21, 28]. In individuals with cardiovascular disease however, enhanced VSMC responsiveness to SNP infusion have been shown following 6–8 weeks of aerobic training [29]. Together this suggests that SNP‐mediated vasodilator responsiveness in non‐active limbs of healthy individuals may show limited detectable adaptation to aerobic training, although whether this reflects high baseline responsiveness, insufficient local hemodynamic stimulus, limited measurement sensitivity, or other factors cannot be determined from the present data. Without underlying vascular dysfunction or external pathophysiological stressors, this upper physiological limit may hinder further upregulation of NO‐mediated vasodilatory mechanisms in VSMCs. This idea reinforces the hypothesis that the potential for additional structural and functional remodeling of the VSMCs may be fundamentally restricted in individuals with healthy vascular function.

In parallel with the unchanged vascular responses to intra‐arterial acetylcholine infusion, we found no effect of the prolonged training intervention on flow‐mediated dilation (FMD). This contrasts with previous reports demonstrating enhanced endothelial function in untrained healthy individuals following exercise training interventions [3, 20]. However, certain methodological aspects of these previous studies may help explain the discrepancies between their findings and ours. For instance, in the study by Argarini et al. [20], participants began with a lower baseline V̇O_2_max (~30 mL·kg^−1^·min^−1^) compared to our cohort (see Table 1), indicating differences not only in aerobic fitness but potentially in baseline vascular function. This lower starting point leaves the possibility that vascular function in these individuals was below optimal and may have allowed greater room for improvements in vascular function. In Clarkson's study [18], V̇O_2_max was not directly assessed; instead, aerobic performance was evaluated using a 1.5‐mile (2.4 km) run time, which improved after the 10‐week training intervention. Moreover, their participants were trained daily, and they combined aerobic (running) and anaerobic (upper‐body strength) exercises, which have been previously shown to promote positive vascular adaptations in the upper limbs [30]. Taken together, differences in baseline fitness, training modality, and overall training volume may partly account for the contrasting results observed across studies.

### 4.2 Prolonged Endurance Training Does not Improve Vascular Function of Non‐Trained Limbs

Consistent with our findings, several studies employing similar methodological approaches have not detected significant changes in vascular function assessed by flow‐mediated dilation or ACh‐induced dilation following 4–8 weeks of endurance training [21, 22]. We hypothesized that extending the training duration to 1 year would elicit measurable and sustained improvements in endothelium‐dependent vascular function, rather than transient or temporary adaptations [31]. However, prolonged endurance training did not alter the vascular response to FMD or ACh infusion at either 1, 4, 6, or 12 months. Together, these contrasting findings underscore the current inconsistency in the literature regarding the efficacy of endurance training in eliciting vascular adaptations in non‐trained limbs of previously untrained populations. Based on the current 1‐year training study, it can be concluded that factors other than duration of the training intervention, such as local hemodynamic stimulus, baseline vascular function, the magnitude and pattern of blood flow and shear stress in the non‐trained limbs, genetic predisposition, age, sex [32], and interindividual variability [33] in endothelial responsiveness, may determine the training adaptations in vascular function in non‐engaged limbs.

### 4.3 Local Versus Systemic Vascular Effects

These findings raise important questions regarding the local versus systemic vascular effects of endurance exercise training, particularly as to whether adaptations are confined to the exercising limb or extend to non‐exercising limbs. Padilla et al. [9] demonstrated that non‐exercising limbs, such as the brachial artery during prolonged leg exercise, exhibit acute vasodilation in response to increased shear stress. The long‐term effect of augmented shear stress in non‐exercising limbs has therefore been proposed as a potential mechanism contributing to systemic vascular adaptations after exercise training. In support of this concept, previous studies have shown that lower limb exercise training can improve upper limb vascular function, with the shear stress response in the upper limb mediating this remote vascular effect [34, 35]. However, more recent evidence suggests that exercise‐induced vascular adaptations may be more region‐specific. For example, 8 weeks of knee‐extension training improved endothelial function primarily in the exercising limb, with limited changes in non‐exercising limbs [22]. Similarly, 6 weeks of high‐intensity exercise training increased vascular function in the popliteal artery, whereas carotid artery distensibility remained unchanged in untrained healthy young subjects [19]. Consistent with these observations, the present study did not demonstrate vascular function improvements at the brachial artery level. The results further support the idea that vascular adaptations to endurance exercise training may be restricted to the exercising limbs.

Despite the absence of vascular adaptations, participants showed a 26.7% increase in V̇O_2_max after 6 months of training, reflecting robust improvements in cardiovascular fitness across the training intervention. Our findings align with prior endurance training interventions in untrained healthy populations and individuals with elevated cardiovascular risk [25, 36, 37]. The improvements in V̇O_2_max highlight the significant effects of endurance training on the cardiovascular system. V̇O_2_max is strongly associated with cardiovascular morbidity and mortality, thus serving as a robust predictor of long‐term cardiovascular health and longevity [38]. As such, this study shows that improvements in cardiovascular health in otherwise healthy participants occur without measurable changes in vascular function.

### 4.4 Limitations

This study utilized a rigorous experimental design, incorporating both invasive and non‐invasive techniques to evaluate VF over 1 year of supervised and well‐structured endurance training. However, several limitations must be acknowledged. First, the small sample size, further reduced by participant dropout, may have limited the power to detect subtle changes in VF. In addition, the unequal sex distribution and the inability to conduct sex‐specific analyses may have obscured potential gender‐dependent adaptations. The limited sample size also likely contributed to increased inter‐individual variability, particularly given the heterogeneous physiological responses to exercise in young, healthy individuals.

Although cycling primarily engages lower‐limb muscles, it can produce systemic increases in shear stress. However, we did not measure the acute systemic effect during training. Including assessments of both trained and non‐trained limbs, at rest and during exercise, would have allowed for a clearer distinction between local and systemic vascular adaptations.

## 5 Conclusion

Contrary to our hypothesis, this study demonstrates that 1 year of supervised endurance training does not lead to measurable improvements in peripheral vascular function in the non‐trained limbs of healthy young individuals. These findings suggest that, in individuals with preserved vascular function, cycle‐based endurance training may provide insufficient local vascular stimulus to induce detectable adaptations in vascular beds not directly engaged during exercise. The absence of systemic vascular adaptation may reflect a region‐specific training effect, with vascular improvements confined to actively engaged limbs and, thus, undetectable in vascular territories not directly involved in exercise. Our results challenge the assumption of widespread systemic vascular benefits from endurance training and offer novel insights into the physiological limits of systemic vascular improvements in healthy, untrained young individuals.

## 6 Perspectives

Future research should determine the conditions under which vascular adaptations extend beyond the actively trained limbs. Studies incorporating exercise modalities that directly increase upper‐limb shear stress, such as arm‐crank exercise or combined upper‐ and lower‐body training, may help establish whether local hemodynamic stimuli are required for remote vascular adaptations. In addition, longitudinal investigations employing repeated vascular assessments throughout the training period could provide insight into the temporal progression of endothelial and vascular smooth muscle adaptations.

The inclusion of populations with impaired vascular health, such as older adults, individuals with cardiometabolic disease, or postmenopausal women, may further clarify whether baseline vascular status influences the likelihood of observing systemic vascular adaptations. Future studies should also examine the potential contribution of sex‐specific factors, circulating vasoactive mediators, and exercise‐induced alterations in autonomic regulation to vascular adaptation across distinct vascular beds.

Methodologically, the integration of vascular imaging, assessments of shear stress exposure, and measurements of endothelial signaling pathways may improve mechanistic understanding of how local and systemic stimuli interact to regulate vascular remodeling during endurance training. Such approaches could help identify the determinants of inter‐individual variability in vascular responsiveness to exercise and guide the development of more targeted exercise prescriptions.

## Author Contributions

Study design: L.G. and M.F. Data collection: M.P.R., L.G., C.S., and J.S.J. Data analyses: M.P.R. and L.G. Data interpretation: M.P.R., L.G., C.S., M.F., A.T.‐E., T.B., J.B., C.L., N.B.N., L.N., and J.S.J. Drafted manuscript: M.P.R., L.G., and C.S. Critically reviewed manuscript: M.P.R., L.G., C.S., M.F., A.T.‐E., T.B., J.B., C.L., N.B.N., L.N., and J.S.J. Approved final version: M.P.R., M.P.R.A., L.G., C.S., M.F., A.T.‐E., T.B., J.B., C.L., N.B.N., L.N., and J.S.J.

## Funding

The Novo Nordisk Foundation grant to Team Danmark (Grant NNF.22SA0078293).

## Conflicts of Interest

The authors declare no conflicts of interest.

## Supporting information


**Figure S1:** Schematic representation of the training intensity zones employed during the 1‐year exercise intervention. Zones are defined as percentages of each participant's individual critical power (CP) and categorized as very light (Z1: 0%–55% CP), light to moderate (Z2: 55%–75% CP), moderate to high (Z3: 75%–90% CP), high (Z4: 90%–105% CP), very high (Z5: 105%–120% CP), and maximal to supra‐maximal intensity (Z6: > 120% CP).

## Data Availability

The data that support the findings of this study are available from the corresponding author upon reasonable request.
