# Supplementary material for: One Year of Endurance Exercise Training Does not Improve Vascular Function in Non‐Trained Limbs in Healthy Individuals
Source: Scand J Med Sci Sports. 2026 Aug 21;36(8):e70354. doi: 10.1111/sms.70354 (PMC13494809; doi:10.1111/sms.70354)
Supplement: Supplementary file 1 [file SMS-36-e70354-s001.zip › Supplementary.pdf]

One year of endurance exercise training does not improve vascular function in non-trained limbs in healthy individuals.

Supplementary Material

Individual training intensity zones were established for each subject's critical power (CP) and programmed using the TrainingPeaks software (https://trainingpeaks.com/) according to a six-zone model (Supplementary Material, Figure 1).

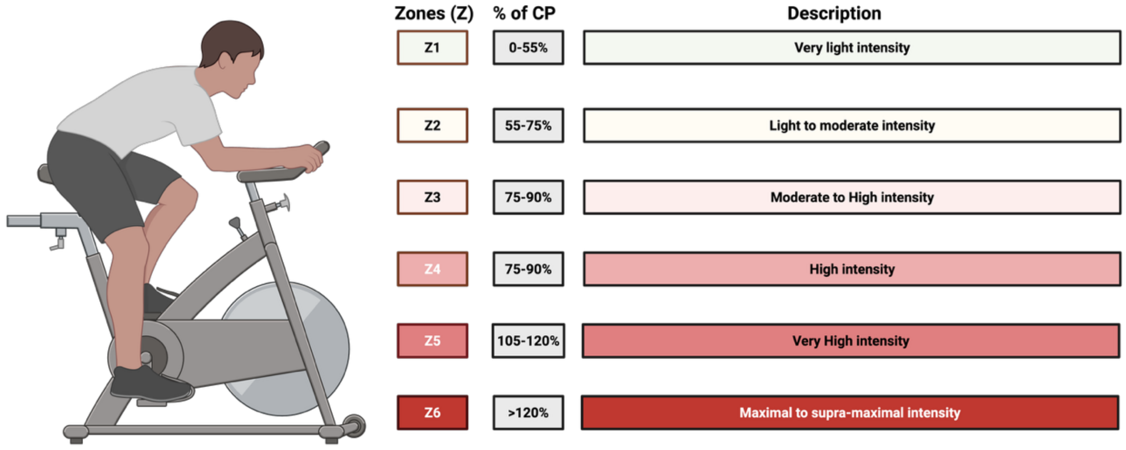

Figure 1. Schematic representation of the training intensity zones employed during the one-year exercise intervention. Zones are defined as percentages of each participant's individual critical power (CP) and categorized as very light (Z1: 0–55% CP), light to moderate (Z2: 55–75% CP), moderate to high (Z3: 75–90% CP), high (Z4: 90–105% CP), very high (Z5: 105–120% CP), and maximal to supra-maximal intensity (Z6: >120% CP).

Training intervention:

For an initial period of two weeks, participants undertook three weekly training sessions lasting 30 to 45 minutes, during which intervals were performed at intensities corresponding to 75–95% of each individual's critical power (CP) to facilitate adaptation to the exercise modality. Subsequently, session durations were increased to approximately one hour, and exercise intensity was raised to include intervals between 95–130% of CP, thereby expediting the transition into high-intensity interval training (HIIT). Between weeks 6 and 10, session durations further advanced to 1–2 hours, accompanied by a progressive increase in weekly high-intensity training volume, which continued systematically throughout the remainder of the 12-month intervention.
